# Phosphate and Ammonium Removal from Wastewaters Using Natural-Based Innovative Bentonites Impacting on Resource Recovery and Circular Economy

**DOI:** 10.3390/molecules26216684

**Published:** 2021-11-04

**Authors:** Miltiadis Zamparas, Grigorios L. Kyriakopoulos, Marios Drosos, Vasilis C. Kapsalis

**Affiliations:** 1 School of Science and Technology, Hellenic Open University, Parodos Aristotelous 18, 26335 Patras, Greece; 2 Photometry Laboratory, Electric Power Division, School of Electrical and Computer Engineering, National Technical University of Athens, 15780 Athens, Greece; gregkyr@chemeng.ntua.gr; 3 Institute of Resource, Ecosystem and Environment of Agriculture (IREEA), Faculty of Biology and Environment, Nanjing Agricultural University, 1 Weigang Road, Nanjing 210095, China; drosos.marios@gmail.com; 4 School of Mechanical Engineering, Industrial Management and Operations Research Sector, National Technical University of Athens, 9 Heroon Polytechniou Street, 15780 Athens, Greece; bkapsal@mail.ntua.gr

**Keywords:** composite material, *f*-MB, wastewaters, resource recovery, fertilizers, circular economy

## Abstract

The research objective of the study is the estimation of a novel low-cost composite material *f*-MB (Fe-modified bentonite) as a P and N adsorbent from wastewaters. Τhe present study aimed at examining the phosphate and ammonium removal efficiency from different types of wastewater using *f*-MB, by conducting bench-scale batch experiments to investigate its equilibrium characteristics and kinetics. The SEM analysis revealed that the platelets of bentonite in *f*-MB do not form normal bentonite sheets, but they have been restructured in a more compact formation with a great porosity. Regarding the sorption efficiencies (Qm), the maximum phosphate sorption efficiencies (Qm) calculated using the Langmuir model were 24.54, 25.09, 26.13, 24.28, and 23.21 mg/g, respectively, for a pH range of 5 to 9. In addition, the maximum NH_4_^+^-N adsorption capacities (Qm) calculated from the Langmuir model were 131.8, 145.7, 168.5, 156.7, and 159.6 mg/g, respectively, for a pH range from 5 to 9. Another important finding of this study is that *f*-MB can recover P from treated wastewater impacting on resource recovery and circular economy (CE). The modified clay *f*-MB performed the phosphate and ammonium recovery rates of 80% and 78.5%, respectively. Finally, *f*-MB can slowly release the largest proportion of phosphate and ammonium ions for a long time, thus extending the application of the *f*-MB material as a slow-release fertilizer and soil improver.

## 1. Introduction

P and N removal and recovery from wastewater have played a vital role in managing ecological and economic concerns, such as the eutrophication of natural waters and a reduction in phosphorus resources [1]. In the European Union, the production of 3.6 Mt N, 1.7 Mt P, and 1.3 Mt K concern the excreta of its citizens. Moreover, the excessive consumption of fertilizers in Europe reaches 11 Mt N, 2.9 Mt P, and 2.5 Mt K [2]. Nowadays, research focuses on the attractiveness and the effectiveness of the treatment of wastewater effluents, especially those containing elemental compositions such as those of phosphorus and nitrogen [3]. Several treatment processes have been widely examined, such as constructed wetlands [4,5,6]—this is an emerging technology that utilizes plants and microbial communities from the rhizosphere, enabling the elimination of a variety of organic and/or inorganic chemical pollutants—aerobic and anaerobic processes, collectively characterized as biological processes [7,8,9], physicochemical processes, indicatively including that of oxidation [10,11], coagulation/flocculation and precipitation [12,13,14], as well as chemical processes such as chemical precipitation with lime [15,16] or NaOH [17]. In most countries, strict legal regulations (e.g., FWD/2000/EU) are followed by P and N discharge in water and wastewater; however, many wastewater treatment methods do not provide the expected level of removal of phosphorus compounds [18].

Resource recovery from wastewaters could be seen as an alternative source for industries and for the agricultural sector that depends on the disposition of each chemical element [18,19,20]. Although the relevance of P removal and recycling is becoming increasingly recognized, the expenses of recovery cannot yet contest in the global market against the mining of P-Rock at this time. [21]. Thus, it is of paramount importance to create efficient and innovative technologies that target phosphate removal and recovery.

**Figure 1:**
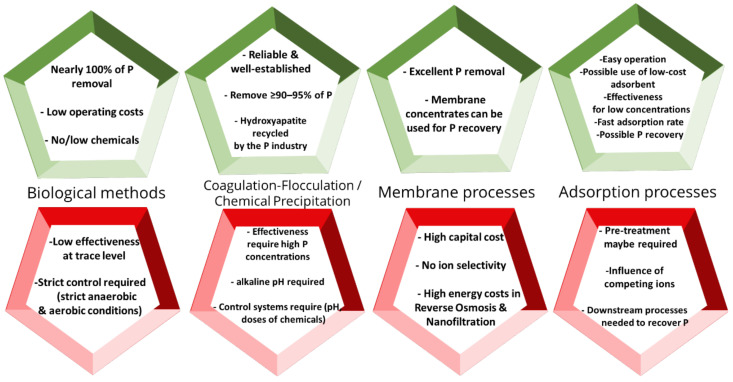
Synopsis of different approaches for P removal and recycling from wastewater (modified from [21,22,23,24,25]).

The existing research in phosphate removal from aqueous solutions and wastewaters has focused on precipitation and biological processes, while the production of struvite and hydroxyapatite are based on phosphate separation and recovery through precipitation and crystallization methods. However, the effectiveness of these methods is determined by the preconditions of highly concentrated phosphate or under the coexistence of ammoniacal nitrogen with phosphate (Figure 1).

Research focus has been also directed on using the method of adsorption for the removal of phosphate and ammonium. The competitive advantages of the adsorption method rely on the severity of effluent standards, the high efficiency of the adsorption method, as well as the utility of P and N loaded as agricultural fertilizers and soil improvements [26]. Moreover, in addition to the technological advantages, adsorption has proven to be simple, easily operative, feasible, cost-effective, and crucially significant due to its environmental friendliness that supports the practical recycling and reuse of phosphorus [27,28], while generating low amounts of sludge [21,29,30]. Moreover, their use has proven to be especially fitting to the target of environmental sustainability in agriculture through leading to the implementation of zero discharge systems [12,31].

P and N adsorption is reached by porous materials with a large surface area and high affinity. Many different types of adsorption materials, such as activated carbon [21], palygorskite [32], La-modified clinoptilolite [33], carbon nanotubes [34], calcite [35], synthetic ferrihydrite [36], bentonite [37], and zeolite [38], have been also effectively established to remove P from wastewaters. However, most of these materials are expensive, sustain low phosphate uptake, or are ineligible for reclaiming and reusing both P and N. Hence, it is essential to develop innovative applicable adsorbents that are highly effective, inexpensive, and eco-friendly for the recovery of P and N from wastewaters.

Even though there are many publications on investigating phosphate removal from wastewaters using clays, there is sporadic literature focus on examining the use of bentonite and its modified clays, in terms of comparing its removal effectiveness under various conditions, as well as under the prospect of removal and recovery phosphate and ammonium simultaneously.

Modified bentonite *f*-MB is a low-cost adsorbent in which Fe and Cu ions are embedded in the interlayer space of natural bentonite. Its basic physicochemical characteristics have been thoroughly investigated in our previous works [39,40]. From a functional point of view, the presence of both positively charged sites, e.g., Fe^3+^, Cu^2+^, surface (+) groups and negatively charged sites (e.g., due to humic acid, HA) render *f*-MB capable of binding both anionic species, e.g., PO_4_^−3^, as well cationic species, e.g., NH_4_^+^. While its performance concerning the adsorption of P (H_2_PO_4_^−^ and HPO_4_^2−^) and N (NH_4_^+^) species from natural eutrophic environments has been studied, its behavior in wastewaters has not been evaluated.

Therefore, in this study, Fe-modified bentonite (*f*-MB) was prepared and applied under different operating conditions to adsorb phosphate anions (H_2_PO_4_^−^ and HPO_4_^2−^) and NH_4_^+^ from wastewaters. Specifically, the present study aimed at examining the phosphate and ammonium removal efficiency from different types of wastewater using *f*-MB under laboratory conditions by conducting batch tests to investigate its equilibrium characteristics and kinetics. These experiments were carried out with synthetic wastewater (SWW) (pure phosphate solutions), real domestic wastewater (RDW), and dairy wastewater (DWW) effluents. Finally, an additional important goal of this work was to determine the application of *f*-MB to recover P from treated wastewater (TWW), acting as an agricultural fertilizer impacting on resource recovery and circular economy (CE).

## 2. Results and Discussion 

### 2.1. SEM

**Figure 2:**
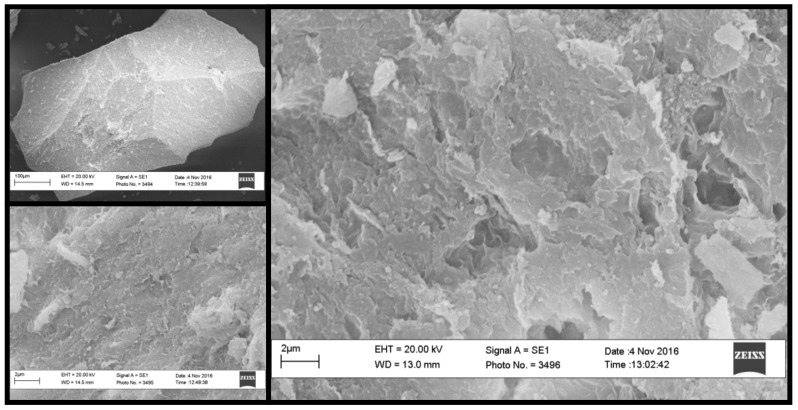
SEM images of innovative modified bentonite *f*-MB.

*f*-MB (Fe-modified bentonite) is an innovative, inexpensive composite material that incorporates Fe and Cu ions and humic acid (HA) into the interstitial space of natural bentonite. Its basic characteristics have been thoroughly investigated in previous works. [39,41,42]. According to Zamparas et al. [43], iron and copper ions have been incorporated in solid crystalline phases, while other phases have been depleted from the pristine clay. Fe and Cu ions are strongly anchored by humic acid’s carboxylates, which are abundant in the HA [43]. Fe and Cu have a great affinity to P and N. Hence, the generated COO-Fe, COO-Cu sites act as coordination sites for P and N [44]. As a result, *f*-MB is an innovative composite adsorbent in which Fe, Cu, HA, and clay lamellas are firmly interconnected. The surface structure of *f*-MB was evaluated using the SEM analytical technique. The SEM images revealed that the platelets of bentonite in *f*-MB have not formed normal bentonite sheets, but have been restructured in a more compact formation with great porosity (Figure 2). In fact, humic acid binding in bentonite and montmorillonite is known to create dense nanoclay structures [45].

### 2.2. Efficiency of f-MB in Phosphate and Ammonium Removal

#### 2.2.1. Synthetic Wastewaters (SWW)

**Figure 3:**
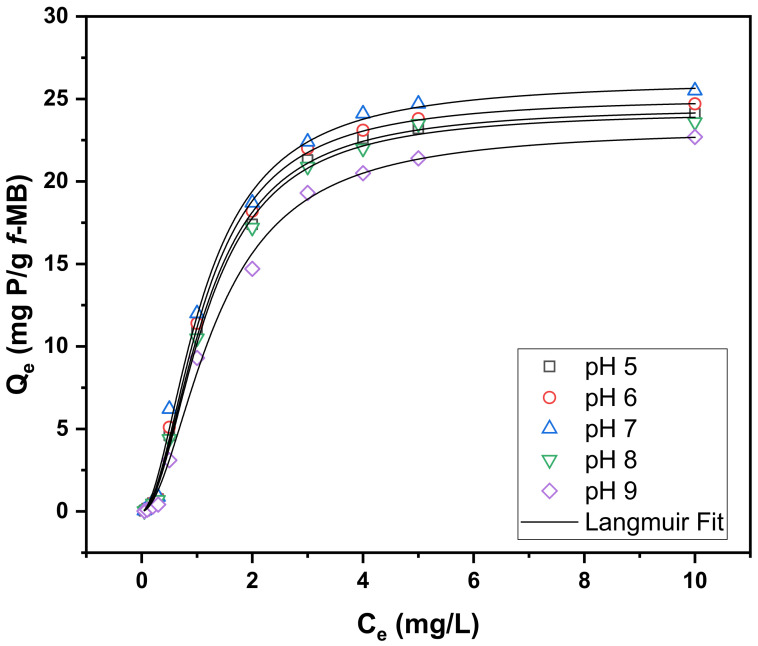
Experimental and calculated (Langmuir model) isotherms fitting of phosphate uptake by *f*-MB at different pH values. Phosphate concentration 0.05–10 mg/L, adsorbent dose 0.2 g, aqueous solution: synthetic wastewater (SWW).

**Figure 4:**
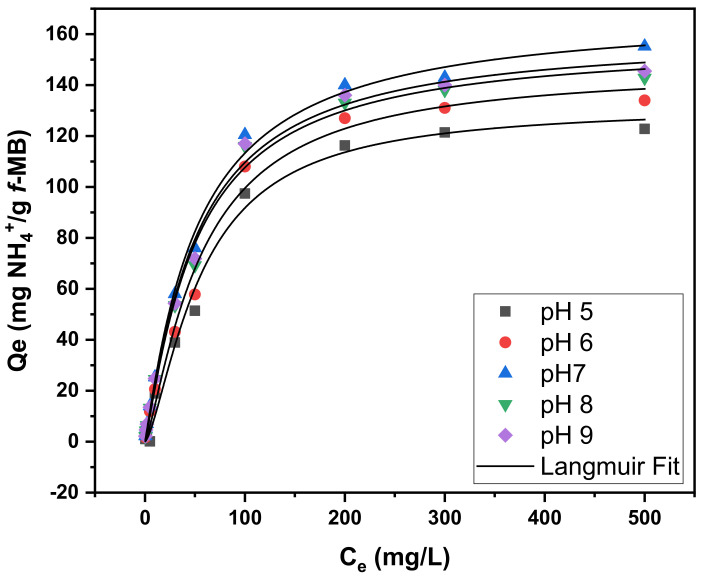
Experimental and calculated (Langmuir model) isotherms fitting of ammonium uptake by *f*-MB at different pH values. NH_4_^+^-N concentration 0.05–500 mg/L, adsorbent dose 0.2 g, aqueous solution: synthetic wastewater (SWW).

The variations in equilibrium adsorption capacities of phosphate and ammonium ions as a function of pH (5–9) are shown in Figure 3 and Figure 4. The phosphate (H_2_PO_4_^−^ and HPO_4_^2−^) adsorption capacity of modified clay *f*-MB in the pH range of 5–7 was greater than the equivalent adsorption capacity at higher pH values, as shown in Figure 3. In the pH range 5–7, the equilibrium adsorption capacity of *f*-MB varied somewhat and decreased in solutions with higher pH values. The pH-dependent increase is due to the enhanced adsorption of formed phosphate anions with pKa 7.2 and the positively charged surface sites of the clay. However, at pH values above 8, the surface of the bentonite becomes negatively charged, leading to a decrease in phosphate adsorption [46]. In general, when the pH exceeds pHpzc, electrostatic repulsion and the increasing competitive effect of OH^−^ ions for the active sites on the sorbent can affect phosphate adsorption [47]. When the pH is lower than pHpzc, the surface of the adsorbent is positively charged, which favors the adsorption of the anions [47]. The material’s point of zero charge (PZC) is 8.8 [43]. This experimental behavior indicates that the positive charges of the material attract phosphate ions (H_2_PO_4_^−^ and HPO_4_^2−^).

As shown in Figure 4, the ammonium adsorption capacity of *f*-MB at the pH of 7 was higher compared to the respective adsorption capacity at lower pH values. The equilibrium ammonium adsorption capacity of *f*-MB changed slightly within the pH range of 7–9 and decreased in solutions with lower pH values (5–6).

Table 1 summarizes the constants of Langmuir adsorption isotherms for phosphate and ammonium on *f*-MB; all values of the regression coefficient (R^2^) were above 0.99, indicating that the model fits the experimental results well.

The maximum phosphate sorption efficiencies (Qm) calculated using the Langmuir model were 24.54, 25.09, 26.13, 24.28, and 23.21 mg/g, respectively, for a pH range of 5 to 9. In addition, the maximum NH_4_^+^-N adsorption capacities (Qm) calculated from the Langmuir model were 131.8, 145.7, 168.5, 156.7, and 159.6 mg/g, respectively, for a pH range from 5 to 9. This pH behavior is consistent with similar experimental studies, stating that the optimal effective reduction was reported at the pH range of 6.0–9.0 [48,49].

#### 2.2.2. Real Wastewaters (RWW)

**Figure 5:**
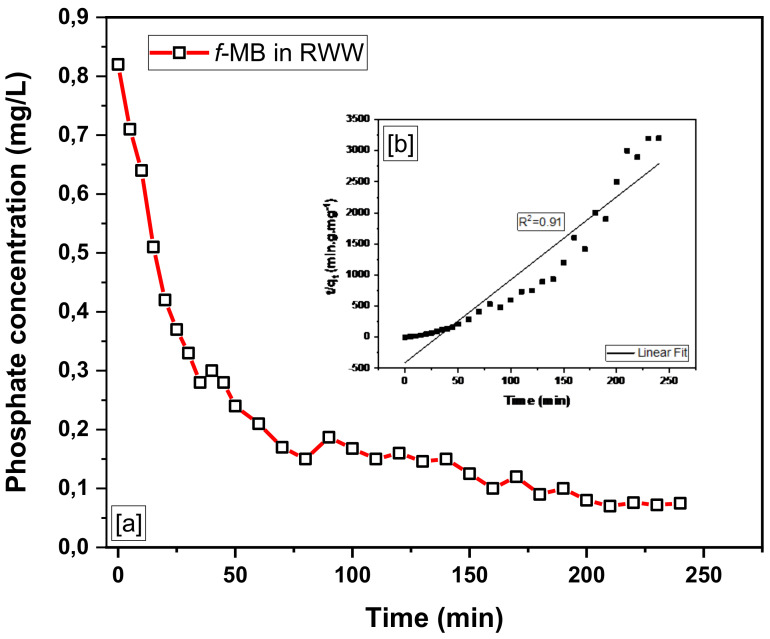
(**a**) Phosphate removal efficiency of *f*-MB in real wastewater. (**b**) Pseudo-second-order kinetics of phosphate-uptake by *f*-MB.

**Figure 6:**
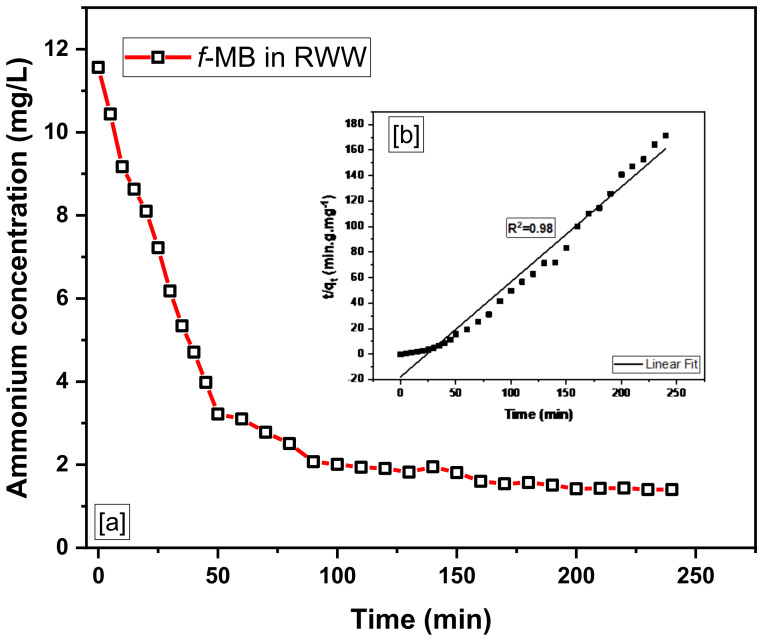
(**a**) Ammonium removal efficiency of *f*-MB in real wastewater. (**b**) Pseudo-second-order kinetics of ammonium-uptake by *f*-MB.

The kinetic data of adsorption of phosphate and ammonium on the modified bentonite *f*-MB are presented in Figure 5 and Figure 6, respectively. The graphs show the amount of phosphate and ammonium adsorbed on *f*-MB as a function of time.

As can be seen in Figure 5, most of the phosphate (H_2_PO_4_^−^ and HPO_4_^2−^) is taken up during the first 30 min of adsorption, and as the contact time increases, the rate of removal slows considerably and is almost negligible after 60 min passage. After 120 min of adsorption time, sorption equilibrium begins to be established. Moreover, most of the ΝH_4_^+^ is adsorbed within 1 h from the start of the batch experiment, and after 5 h, the isotherm reaches equilibrium (Figure 6). Adsorption rates are high at the beginning of the experiment due to the rapid filling of active sites (boundary layer diffusion) and gradually decrease due to intraparticle diffusion processes. In similar experimental studies, high adsorption rates have also been reported regarding the high sorption capacity of Phoslock^ΤΜ^ [50].

To gain valuable insight into the adsorption mechanism and kinetics to sort out the rate-controlling step of the adsorption process, a pseudo-first and pseudo-second-order model were used to examine the experimental kinetics data of phosphate (H_2_PO_4_^−^ and HPO_4_^2−^) and ammonium (ΝH_4_^+^) adsorption on *f*-MB. The pseudo-second-order model provided better agreement.

Indeed, linear plots of t/qt vs. t in Figure 5b and Figure 6b demonstrate the system’s suitability for the pseudo-second-order model. With correlation values better than 0.90, the pseudo-second-order kinetic equation was well associated with the experimental data for phosphate and ammonium adsorption.

#### 2.2.3. Dairy Wastewaters (DWW)

**Figure 7:**
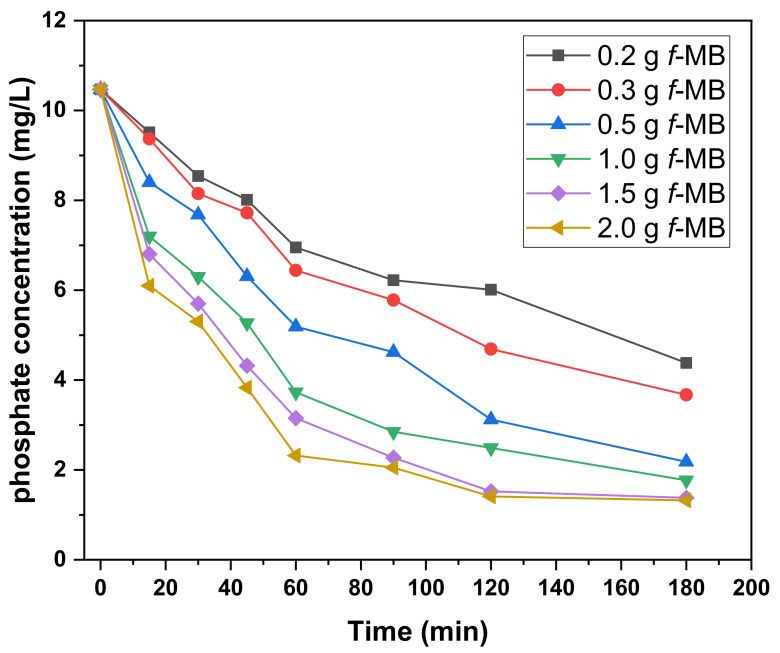
Phosphate adsorption from DWW exposed to different amounts of *f*-MB (0.2–2 g). Phosphate initial concentration: 10.47 mg/L.

Phosphate adsorption results from the second cheese whey wastewater are presented in Figure 7. It can be seen that the most rapid adsorption was obtained at dosages of 1.5 and 2.0 g. Phosphate was rapidly removed from the solution after 30 min, with 35 and 41.7% removal, respectively. Moreover, an uptake of 86.8% and 87.4% was achieved 180 min after the addition of *f*-MB using 1.5 and 2.0 g, respectively. However, the dose of 0.2 mg showed slower adsorption, with 18.4% and 58.16% removal after 30 and 180 min, respectively.

Compared to domestic wastewaters (RWW), the low phosphate adsorption capacity of *f*-MB in the secondary cheese whey wastewaters (DWW) is probably due to the low pH values prevailing in these samples.

#### 2.2.4. Regeneration and Reuse *f*-MB for P and N Removal from Wastewaters

**Figure 8:**
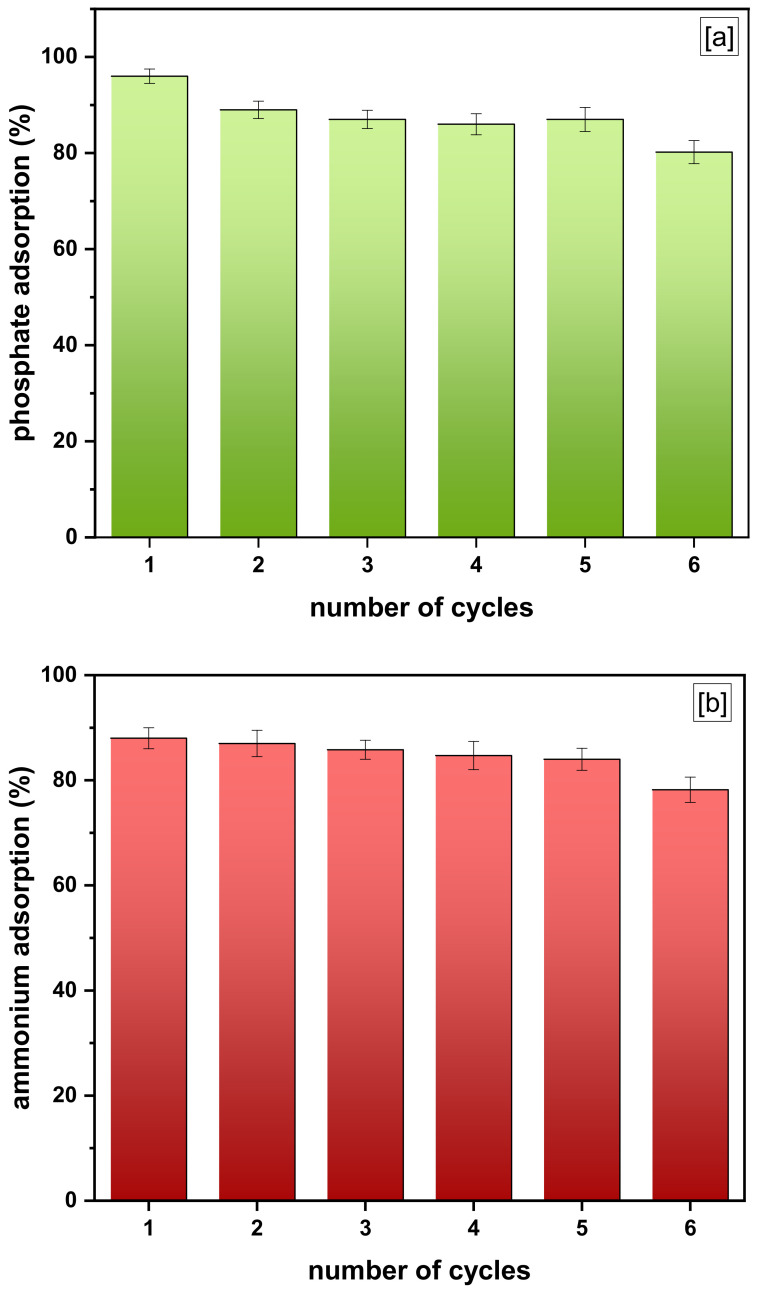
Regeneration and reuse studies of *f*-MB for the removal of (**a**) phosphate and (**b**) ammonium.

The reusability study helps illustrate and explain the regenerating capability of the *f*-MB, being also an important sorbent for practical applications in agriculture and environmental remediation purposes. The regenerated *f*-MB was subjected to six cycles of adsorption/desorption using a base solution as desorbent. The results displayed that NaHCO_3_ (0.4 M) was a suitable eluent for the regeneration of *f*-MB (Figure 8a). The phosphate adsorption slightly decreased from 96 to 80% for *f*-MB after six consecutive cycles. In addition, as demonstrated in Figure 8b, the NH_4_^+^-N adsorption efficiency of *f*-MB was sufficiently maintained from the first to the sixth cycle. Specifically, the NH_4_^+^-N adsorption declined from 88 to 78.2%. Thus, the experimental results showed that *f*-MB can be effectively regenerated by NaHCO_3_ treatment using the adsorption/desorption method.

#### 2.2.5. P and N Release Experiments for Sustainable Applications of *f*-MB

**Figure 9:**
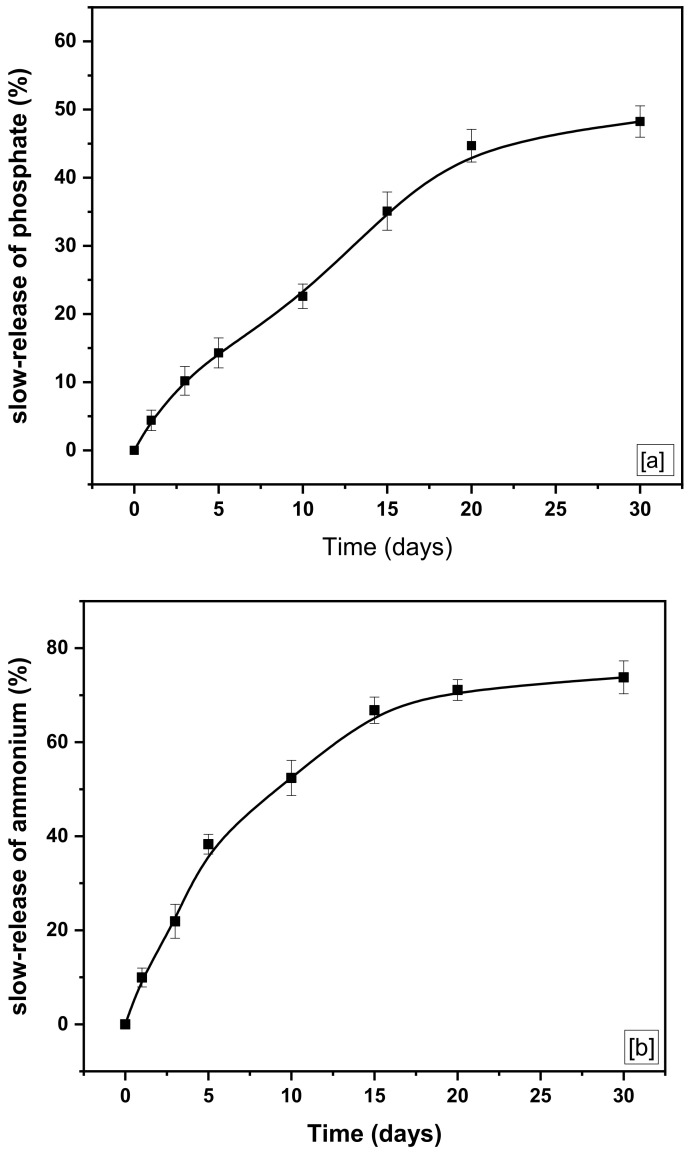
(**a**) Phosphate and (**b**) ammonium release from *f*-MB into the soil.

According to previous works [39,51,52], *f*-MB is suitable for controlled agricultural soil fertilization because it allows the gradual release of nutrients such as N and P, which can promote high crop yields. Therefore, P and N release studies were conducted to determine its appropriateness for direct use as a fertilizer and soil improver. Initially, the release experiments were carried out using P- and N-loaded *f*-MB, and the results showed that phosphate (H_2_PO_4_^−^ and HPO_4_^2−^) and ammonium (ΝH_4_^+^) release was notably slow, and final release equilibrium was reached after 30 days, at which time 48.25% and 69.7% of the absorbed phosphate and ammonium had been released, respectively (Figure 9). Specifically, the release pattern of phosphate from *f*-MB involves the stages below (Figure 9a).

The first stage released about 14.3 wt% of the phosphate ions (H_2_PO_4_^−^ and HPO_4_^2−^) within 5 days. The second stage (from 5 to 15 days) is confirmed by a slower rate of P release, with approximately 20.8 wt% of the phosphate released within these 10 days. The third step lasts 15 days (from day 15 to day 30) to attain the equilibrium of gradual release, in which 13.5 wt% of phosphate was released within the aforementioned period. This step involves the slow release of nutrients to provide plants over a longer period [53].

Figure 9b shows the release rate of ammonium (ΝH_4_^+^) from the modified bentonite *f*-MB. Based on Figure 9b, it can be inferred that a noteworthy percentage of the initially adsorbed ΝH_4_^+^ is released into the soil. Specifically, the release pattern of ΝH_4_^+^ from *f*-MB involves the stages below (Figure 9b). A significant initial ΝH_4_^+^ release was observed in the first phase, with 38.3 wt% released within five days.

At the initial stage, a high initial ΝH_4_^+^ release rate was observed, of which 38.3 wt% of ΝH_4_^+^ was released within 5 days. The next stage (from 5 to 15 days) is confirmed by a slower rate of release, of which about 28.5 wt% of ammonium was released within these 10 days. The third stage is extended from 15 days (from 15th to 30th day) to achieve the slow release equilibrium, in which about 7.0 wt% of ΝH_4_^+^ was released within the aforementioned period. The first significant ammonium leaching is qualified by the fact that NH_4_^+^ adsorbed by the *f*-MB is immediately released into the soil via ion exchange between H^+^ in soil and NH_4_^+^ adsorbed in the material’s matrix. This behavior indicated that the *f*-MB can slowly release the largest proportion of phosphate and ammonium ions for a long time, thus extending the application of the *f*-MB material as a slow-release fertilizer and soil improver.

Many works have been carried out to estimate the desorption of phosphate and ammonium in aqueous solutions, showing low desorption rates. However, these results can be well described by the properties of the aqueous solution used in the desorption tests [54,55] since the solution depletes any ion exchange capacity to release and replace ΝH_4_^+^ ions. Thus, the results are more reliable when the experiments are performed in the presence of soil, studying the release of nutrients that have been adsorbed on the materials that will be used as future fertilizers.

## 3. Materials and Methods

### 3.1. Reagents

Purified water Milli-Q produced by the Millipore Academic System (Millipore Corporation, Molsheim, France) with a conductivity of 18.2 S was used to prepare all solutions. Composite bentonite (*f*-MB) was synthesized as is previously stated in Zamparas et al. [43,56].

### 3.2. Phosphate and Ammonium Adsorption Batch Experiments

#### 3.2.1. Synthetic Wastewater (SWW)

Synthetic (inorganic) wastewater (SWW) was prepared by drying commercial potassium dihydrogen phosphate (KH_2_PO_4_) and ammonium chloride (NH_4_Cl) at 104 °C for 24 h, followed by dilution with deionized water (DW). A total of 0.2197 g KH_2_PO_4_ was dissolved in 1.0 L deionized water to prepare a 50 mg/L phosphate stock solution, and 3.819 g NH_4_Cl was dispersed in 1.0 L deionized water to make a 1000 mg/L ammonium stock solution, and dilutions of the stock solutions were employed in subsequent experiments.

Adsorption isotherm data of phosphate and ammonium on *f*-MB in different initial concentrations ranging between 0.05–10 mg/L and 0.05–500 mg/L were studied using optimized conditions: 50 mL aqueous solutions, adsorbent dose 0.2 g, and stirrer rotation 200 r/min. The experiments were carried out at room temperature (25 ± 1 °C), and pH varied from 5 to 9 for 4 h. For the pH-edge experiments, a buffer system of 10 mM MES: (*N*-morpholino-ethanesulfonic acid), HEPES: (4-(2-hydroxyethyl)piperazine-1-ethanesulfonic) acid was used in all of the samples [43,57]. This system presented a significant buffering capacity at a range of pH 5–8.5 with an average deviation from the adjusted pH value of less than 5%. Screening experiments indicated that under the conditions of our experiments, the buffer molecules caused no interferences on the adsorption phenomena [57]. Prior to starting the experiment, the pH was adjusted from 5 to 9 at 25 ± 1 °C using 0.1 M HCl and NaOH solutions. All experiments were carried out in duplicate.

#### 3.2.2. Real Domestic Wastewaters (RDW)

Phosphate and ammonium adsorption kinetics in domestic (anaerobically digested effluent wastewater, DW) treated effluents were carried out on effluent sources from a wastewater treatment plant (Patras, Greece). The Wastewater Treatment Plant of Patras (Rion) was established in 1990 and it produces between 1000 and 2000 m^3^/day of sewage [58]. The plant primarily processes wastewater from the city, as well as some hospital wastewater from University General Hospital (Patras, Greece) [59,60]. During the dry season, the treated effluents are disposed of in a 20 hectare area of eucalyptus and poplar trees. During the rainy season, they are discharged into a stream before being disposed of in the Patraikos Gulf [58]. The samples were immediately taken to the laboratory and their physicochemical parameters were determined (Table 2). The EPA Standard Methods for the Examination of Water and Wastewater [61] were used to determine the physicochemical parameters, as well as the phosphate and ammonium concentrations (Table 2). 

**Figure 10:**
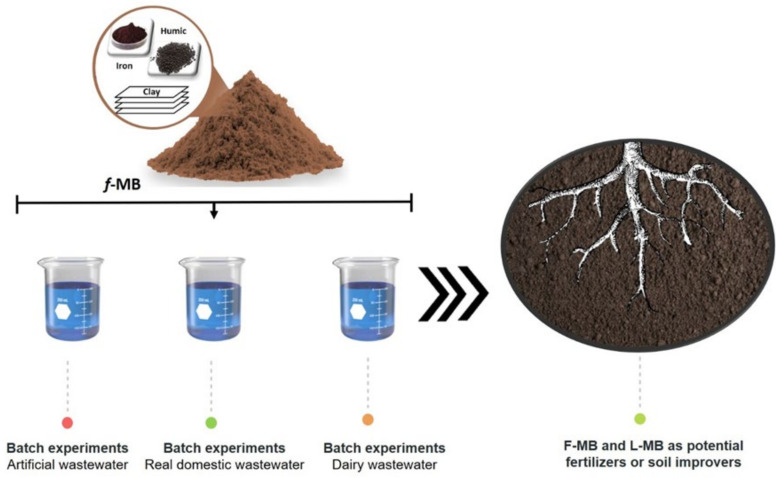
Schematic diagram of the set-up of the batch sorption experiments under different effluents and the application of adsorbents as fertilizers. Source: author’s own study.

The kinetic data of phosphate and ammonium adsorption on *f*-MB were examined under specific conditions for contact times ranging from 15 to 240 min: pH 7.8, phosphate concentration 0.82 mg/L, ammonium concentration 11.56 mg/L, adsorbent dosage 0.2 g, stirrer rotation 200 r/min, and 25 ± 1 °C temperature, Figure 10.

#### 3.2.3. Dairy Wastewaters (DWW)

Phosphate adsorption against different amounts of *f*-MB in dairy wastewater was carried out on dairy wastewater (Aigio, Western Greece) passed through a mechanical slurry solid/liquid separator and a primary settling pond. The physicochemical parameters of dairy effluents such as pH, dissolved oxygen, phosphate, and NH_4_^+^ concentrations are collectively listed in Table 2. A total of 100 mL of dairy effluent with an initial phosphate concentration of 10.47 mg/L were added into borosilicate conical flasks containing *f*-MB in different amounts, ranging from 0.2 to 2.0 g of *f*-MB. The suspension was centrifuged and the concentration in the supernatant (Ce) was determined by molybdate blue spectrophotometry [62].

Phosphate (H_2_PO_4_^−^ and HPO_4_^2−^) ions were calculated from the concentration difference between the initial (C_0_) and equilibrium concentrations. Blank samples without absorbents were performed and monitored as controls. All experiments were perpetrated in duplicate.

### 3.3. Desorption Studies

#### 3.3.1. Regeneration and Reuse *f*-MB for P & N Removal from Wastewaters

In the regeneration procedure, the 0.2 g *f*-MB, used for co-adsorption of phosphate and ammonium, was collected, oven-dried for 24 h, and then placed in a 150 mL flask containing 50 mL of elution solution (eluent: sodium bicarbonate NaHCO_3_ 0.4 M). Regeneration experiments were carried out by using the standard methodology described by Wu et al. [63]. The material was then washed with Milli-Q water, dried, and reused for the next cycle. The regeneration experiment was repeated for six continuous cycles.

#### 3.3.2. Slow-Release P in Soil

To examine the release behavior of phosphate (H_2_PO_4_^−^ and HPO_4_^2−^) and ammonium (NH_4_^+^) ions from *f*-MB in soil, a soil experiment was conducted following Wang et al. [43]. A total of 0.2 g of novel adsorbent *f*-MB was enclosed in a nonwoven polypropylene mesh bag, buried 5 cm above the soil, and packed in a PVC pot filled with 100 g of dry loam soil (sieve size: 0.2 mm) at room temperature. The pH was measured from water extracts of the soil prepared from a 1:2.5 soil:water suspension. The pH was 6.1 and was measured using a pH meter. Throughout the experiment, the moisture content of the soil samples was maintained at 30 wt%, and the water content ratio of the soil was weighed and an additional amount of tap water was added as needed. After 1, 3, 5, 10, 15, 20, and 30 days, the net bags were collected and dried at room temperature to determine the content of phosphate and ammonium. P and N concentrations were evaluated by Olsen’s method and the indophenol blue method, respectively.

### 3.4. SEM Analysis

The *f*-MB sample morphology was investigated by a scanning electron microscope (SEM ZEISS EV040, Carl Zeiss QEC GmbH, Ostfildern, Germany). A freeze-dried sample was placed on a stub and sputter-coated with gold palladium (AGAR sputter COATED B 7340). SEM observations were conducted at 10.13 K magnification, with an accelerating voltage of 20.00 kV, at a 10 mA current and using 8.5 mm < WD < 12.5 mm as a focal length.

## 4. Conclusions

This work is part of an ongoing effort to develop innovative approaches in phosphate and ammonium removal, supporting resource recovery and circular economy. In this study, the performance of a novel modified bentonite (*f*-MB) for the uptake of phosphate and ammonium from different types of wastewaters was studied through a number of batch experiments. The findings of the work cited in this publication were:

▪ The natural-based innovative *f*-MB was highly efficient for simultaneous P and N removal from wastewaters at a wide range of pH values (5–9). Compared with other various materials commonly used for phosphate adsorption from wastewaters, *f*-MB exhibits higher adsorption capacity (Table 3). In addition, the simultaneous removal of P and N by Fe-modified bentonite is an additional advantage over other materials that are unable to adsorb phosphate and ammonium ions simultaneously and are usually selective concerning P or N (Table 3). For example, the material shows more than three times the adsorptive capacity of La-modified bentonite and over twice the adsorptive capacity of Fe-Al pillared bentonite (Table 3). From the overview in the literature, it can be concluded that our material shows high efficiency, which is in the same order of magnitude as materials, such as iron oxide/zeolite, while it is significantly superior to most materials mentioned in the literature (Table 3). The clay used appeared to be quite efficient in adsorbing phosphate and ammonium under different types of wastewaters, such as municipal/domestic wastewaters and dairy wastewaters.
▪ *f*-MB can be effectively regenerated by NaHCO_3_ treatment using the adsorption/desorption method. The modified clay *f*-MB achieved the phosphate and NH_4_^+^ recovery rates of 80% and 78.5%, respectively.
▪ *f*-MB can slowly release the largest proportion of phosphate and ammonium ions for a long time, thus extending the application as a slow-release fertilizer and soil improver.

Modified bentonite *f*-MB can be effectively utilized both as a filter medium in filter-based systems and as a bed medium in constructed wetlands. The operation of this promising environmental remediation agent becomes saturated with phosphate and ammonium after a period of use, resulting in decreasing the removal efficiency of P and N; thus, it can be used as an immediate source of P and N fertilizer for irrigated crops, or it can be regenerated after the adsorbed nutrients have been removed. The proposed technology can partially overcome the perceived future shortage of phosphate global reserves. Then, after phosphate removal, the generated low-cost sorbents can enrich phosphate-poor plants by directly applying these sorbents to lands. Moreover, *f*-MB sustains the presence of humic acid that can produce additional benefits, such as an organic-increasing content, an acid-ameliorated soil, and soil structure stability [74]. Finally, the proposed trials have to be extended by using continuous mode column trials that practically resemble real operating systems (forthcoming publication). Removal recovery of phosphate by sorption and the subsequent production of fertilizers should be prioritized in future research of full-scale plants.

## Figures and Tables

**Table 1:** The constants of Langmuir and R^2^ for adsorption of phosphates and ammonium on novel *f*-MB at pH 5–9.

	Model	Equation	Parameter	pH 5	pH 6	pH 7	pH 8	pH 9
	Langmuir	$\begin{array}{l} q_{e}=\frac{bq_{m}C_{e}}{1+bC_{e}} \end{array}$	q_m_	24.54	25.09	26.13	24.28	23.21
**H_2_PO_4_^−^/HPO_4_^2−^**			b	0.75	0.79	0.83	0.72	0.56
			R^2^	0.99	0.99	0.99	0.99	0.99
			q_m_	131.8	145.7	168.5	156.7	159.6
**NH_4_^+^**	Langmuir		b	0.002	0.005	0.013	0.011	0.011
			R^2^	0.99	0.99	0.99	0.99	0.99

*C_e_* (mg/L) and *q_e_* (mg/g) are the equilibrium adsorbate concentrations in the aqueous and solid phases, *q_m_* (mg/g) is the maximum adsorption capacity, and *b* is the Langmuir adsorption equilibrium constant.

**Table 2:** Physicochemical parameters of the domestic wastewater used in the batch experiments.

Parameters	RDW	DWW
Total solids (%)	3.0	5.9
Phosphate (mg/L)	0.82	10.47
Ammonium (mg/L)	11.56	120.14
BOD (mg/L)	145	58
COD (mg/L)	188	64
Calcium (mg/L)	52.3	-
Magnesium (mg/L)	16.4	-
Potassium (mg/L)	17.7	-
Sodium (mg/L)	164	-
Temperature (°C)	20	23.4
pH	7.8	4.12

**Table 3:** Comparison of phosphate adsorption capacity (qm) of tested material reported in the literature.

Adsorbent	Qm Phosphate (mg/g)	Qm Ammonium (mg/g)	References
Modified palygorskite-bentonite clay	1.74	12.87	[64]
Hydroxy-Fe-Al pillared bentonite	10.5	-	[65]
Lanthanum-modified zeolite	6.6	-	[66]
Modified bentonite	-	5.85	[67]
Al-bentonite	12.7	-	[65]
Iron oxide/zeolite	38.91	3.74	[68]
Natural halloysite	-	1.66	[69]
La-modified clinoptilolite	8.3	-	[66]
Natural Ca-bentonite	0.5	-	[70]
Calcite	6	-	[71]
Natural bentonite (Algerian)	-	19.01	[72]
Potassium clinoptilolite	6.8	29.0	[73]
Fe-modified bentonite *f*-MB	26.13	168.5	This study

## Data Availability

Not applicable.

## Sample Availability

Samples of the compounds are not available from the authors.
